# Telephone-based aftercare groups for family carers of people with dementia – results of a mixed-methods process evaluation of a randomized controlled trial

**DOI:** 10.1186/s12913-023-09579-1

**Published:** 2023-06-15

**Authors:** Susanne Lessing, Ruth Deck, Martin Berwig

**Affiliations:** 1 Clinic for Cognitive Neurology, Medical Faculty, University of Leipzig, Liebigstr. 16, 04103 Leipzig, Germany; 2 Institute for Social Medicine and Epidemiology, University of Lübeck, Ratzeburger Allee 160, 23538 Lübeck, Germany; 3 Institute for General Practice, Medical Faculty, Otto-Von-Guericke-University of Magdeburg, Leipziger Straße 44, 39120 Magdeburg, Germany; 4 German Centre for Neurodegenerative Diseases (DZNE), Stockumer Straße 12, 58453 Witten, Germany; 5 School of Nursing Science, Faculty of Health, Witten/Herdecke University, Alfred-Herrhausen-Straße 50, 58448 Witten, Germany

**Keywords:** Family carers, Dementia, Rehabilitation, Telephone-based aftercare groups, Process evaluation, Mixed methods

## Abstract

**Background:**

Caring for a relative with dementia can be demanding and time-consuming. It is not uncommon for them to be overburdened and overworked, which can lead to symptoms of depression or anxiety disorders in 2/3 of cases. One possibility for treating family carers who have these issues is special medical rehabilitation (rehab). However, studies have shown that while such rehab is effective, it is not sustainable.

To increase the sustainability of rehab for this target group, structured telephone-based aftercare groups were implemented in the present study. A process evaluation was conducted focusing on the acceptability of the aftercare programme and its perceived benefits by the participating family carers and group moderators.

**Methods:**

The process evaluation was embedded in a longitudinal randomized controlled trial and followed a mixed methods approach. Quantitative process data were collected using protocols and structured brief evaluations regarding the telephone-based aftercare groups. To assess the acceptability of the aftercare groups as well as their subjective evaluation by the participants, qualitative process data were collected through two longitudinal telephone-based interviews with a subsample of family carers as well as a focus group interview with the group moderators.

**Results:**

Telephone-based aftercare groups provide acceptable and supportive experiences, and they are shown to be practicable. The content structure and the procedure of the group sessions could be easily implemented in everyday life after inpatient rehab. The topics addressed with each patient were met with a consistently positive response. Learning from the other group members and sharing a bond based on the experience of caring for a relative with dementia were evaluated as positive outcomes in the group. The universality of suffering as a central effective factor of group psychotherapy also played a decisive role in this telephone-based support group format for a shared bonding and strengthening experience in the groups and thus for their effectiveness.

**Conclusion:**

Telephone-based aftercare groups for family carers of people with dementia are a useful and acceptable tool in the context of rehab aftercare. This location-independent aftercare programme could be adapted for other indications, focuses or topics in everyday care.

**Trial registration:**

German Clinical Trials Register: DRKS00013736, 14/05/2018.

**Supplementary Information:**

The online version contains supplementary material available at 10.1186/s12913-023-09579-1.

## Background

Relatives who care for a person with dementia are exposed to extensive demands and stresses [1–4]. The stress experienced by carers is particularly high if they do not receive support in caring for their relative with dementia [5]. It is often difficult for family carers to take advantage of assistance because relief services such as day care, outpatient care or individual counselling are not available or are inadequate and/or knowledge about these offers and their financing (e.g., through care legislation) is lacking [6]. Care and nursing are time-consuming and bring special challenges to the carer due to dementia symptoms such as memory loss, changes in personality, challenging behaviour, loss of independence, and a high need for care, which pose an increased risk of physical and mental health problems for carers [7]. Two-thirds of all family carers in Germany, including those caring for a person with a condition other than dementia, experience mental health problems, like symptoms of depression or anxiety [8]. Medical rehabilitation (rehab) can help support family carers with mental health problems and restore their health. This was shown by a study that evaluated a rehab treatment in northern Germany specifically designed for family carers of people with dementia. However, the health-related and psychosocial effects of this treatment were not sustainable and could no longer be detected after six months [9]. Thus, aftercare is of particular importance, especially for rehabilitants who care for a relative with dementia. In the medical health services landscape in Germany, there are a large number of established aftercare programmes, such as training and education programmes or counselling services offered by the German Pension Insurance or the health insurance fund, which contain various components to strengthen competence and health. For family carers, however, it is often difficult to organize care for the person with dementia and to spare time for aftercare measures. The Talking Time REHAB project was created to tackle this difficulty in transferring rehab effects to the home environment and showed that carers can be supported in a consolidated manner by telephone-based (and thus location-independent) aftercare groups [10]. This project idea is also more comprehensively described in the related study protocol [11]. A meta-analysis shows that telephone -based aftercare can help to increase the sustainability of rehab, especially if there is a high intensity of care [12]. However, the included studies used predominantly dyadic concepts. In contrast, a telephone-based group setting was chosen for the aftercare of family carers in Talking Time REHAB, and the aftercare was conducted in the context of a telephone conference. Groups can prevent social isolation and present the possibility of exchange and learning from each other, which is also used in theme-centred interaction (TCI) according to Ruth Cohn [13]. Therefore, TCI was used as the conceptual background for the aftercare groups. Formally, the aftercare groups were based on telephone-based groups in conjunction with the home visiting programme for family carers of people with dementia developed in the USA (Resources for Enhancing Alzheimer`s Caregiver Health II, REACH II) as an intervention component for social support [14, 15].

The Medical Research Council (MRC) framework provides a well-established guide for the development of complex healthcare interventions [16, 17]. The revised 2008 version of this framework [16, 18] emphasizes for the first time that conducting a process evaluation is highly recommended to understand processes and obtain explanations when interventions fail or have unforeseen consequences.

Following this recommendation [16, 18], a comprehensive process evaluation of the telephone-based aftercare groups was conducted as part of the overall evaluation of the Talking Time REHAB project, the results of which are presented in this manuscript. The aim was to answer the question of which structures and processes contribute to the successful implementation of the aftercare offer and to what extent the participants of the telephone-based groups experienced these groups as strengthening their daily care routine.

## Materials and methods

### Study design

To investigate the long-term effects of telephone-based aftercare groups, a randomized controlled prospective longitudinal study was conducted at one rehab centre. The process evaluation of the telephone-based aftercare groups from that study was integrated into this study design [10] and followed a triangulated "concurrent" mixed-methods approach in which quantitative and qualitative survey and evaluation methods were applied independently of each other and the results were combined at the end [19]. The methodological details of the collection, analysis and results of the qualitative process data have been published elsewhere in the German language [20] but have not yet been presented and discussed in the context of the mixed-methods approach of this process evaluation. This will be addressed in the present article.

### Intervention

**Fig. 1:**
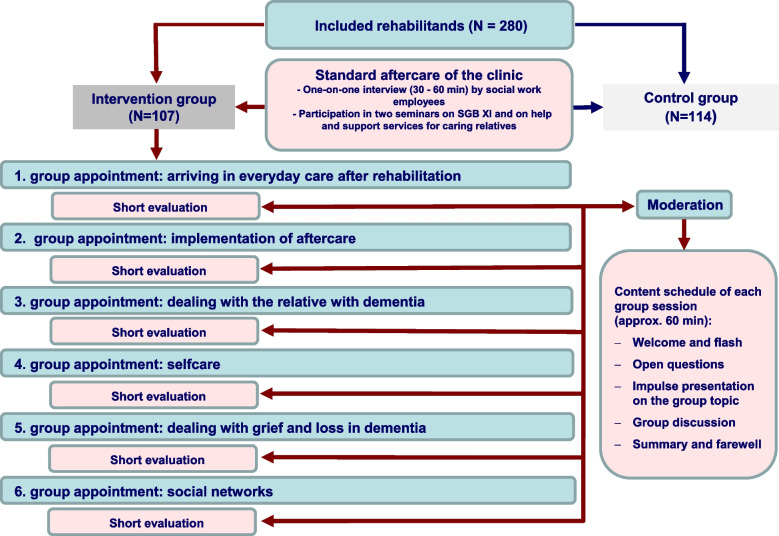
Procedure of the telephone-based aftercare groups (translated from [20]). Legend: *N* Number

The schedule of the telephone-based aftercare groups with the topics of the individual sessions is shown in Fig. 1. The groups were designed to have five family carers in each and followed a fixed scheme that was methodologically based on TCI [13]. Moderation was provided by two social workers from the rehab clinic. The participants were handed out an accompanying folder designed for the group discussions containing the themes overview, notes on the procedure, suggestions for more in-depth literature, and feedback sheets. A detailed description of the conceptual background and the intervention components can be found in the study protocol [11] and the publication on the results of the effect evaluation of the Talking Time REHAB project [10].

#### Intervention group

At the end of the rehab stay, the participants in the intervention and control groups received a portfolio of aftercare recommendations, such as useful support services, as well as recommendations for self-care in everyday nursing and caregiving. The aftercare recommendations were discussed intensively at the end of the rehab programme. The participants in the intervention group were also given the dates of the telephone-based aftercare group sessions during this discussion [10].

#### Control group

The family carers of the control group received only a portfolio of aftercare recommendations that were adapted to the individual situation of the respective family carer at the end of rehab. These recommendations have been self-implemented and were not accompanied by any aftercare measurement. During the intervention period, they continued with their usual activities or on-site services at home, without restriction (usual care).

### Data collection

Quantitative process data were collected during and after each telephone-based group session, and qualitative process data were collected after the entire intervention was completed.

#### Quantitative process data

**Fig. 2:**
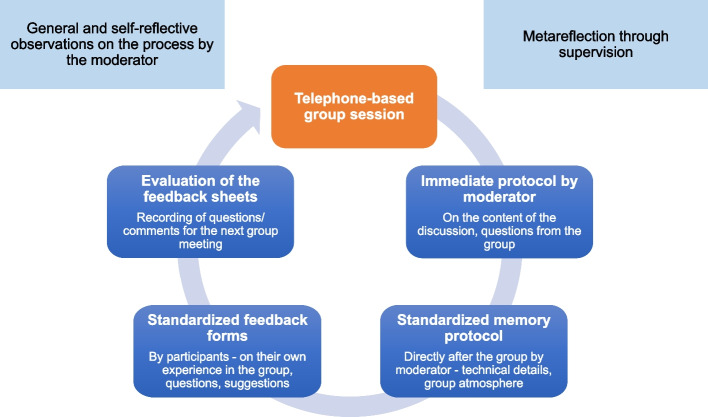
Procedure of the formative evaluation

The quantitative process data were collected with the help of immediate and memory protocols, which the moderators created during or after the group sessions, as well as structured short evaluations (feedback sheets) that the participants filled out after the end of the aftercare group session. During the intervention phase, the immediate and memory protocols and the feedback forms were initially used as instruments for formative evaluation [21]. In this context of formative evaluation, a meta-reflection of the moderators by external supervision as well as a written self-reflective observation of the intervention process by the moderators themselves took place across the process and with a focus on group dynamics. Formative evaluation should identify the need for possible adaptation of the intervention and should optimize the quality of the intervention (see Fig. 2).

1. ***Immediate protocols.*** The meeting minutes were immediately recorded by the moderators during the telephone group session and were not evaluated separately. They contained notes on the conversation and served as a memo for the moderators in case of questions during the telephone sessions.
2. ***Memory protocols.*** The memory log was created immediately after each session and included technical details (e.g., voice quality), data on participation, adherence to formal procedures, content delivery, and an assessment of the group atmosphere and dynamics (for the blank form of the memory protocol, see Additional file 1).
3. ***Feedback sheets.*** Each session of the telephone-based aftercare groups was evaluated by the participants using a feedback sheet (for the blank form of the feedback sheet see Additional file 2). The questions were related to content (e.g., relevance of the topics), their own experience in the group (e.g., well-being, involvement in the group discussion, relief from the group), moderation (e.g., kindness, attention) and aftercare recommendations (e.g., the implementation of recommendations from the rehab, existing need for support). Furthermore, the family carers had the opportunity to communicate questions or suggestions to the moderator via feedback sheets, which were incorporated into the formative process. The sheets were first sent by the participants to the moderator, who used them to record information for the subsequent group session (see Fig. 2). After the end of the entire intervention, they were sent together with the memory protocols to the Institute for Social Medicine and Epidemiology (ISE) of the University of Lübeck for summative evaluation.

#### Qualitative process data

**Fig. 3:**
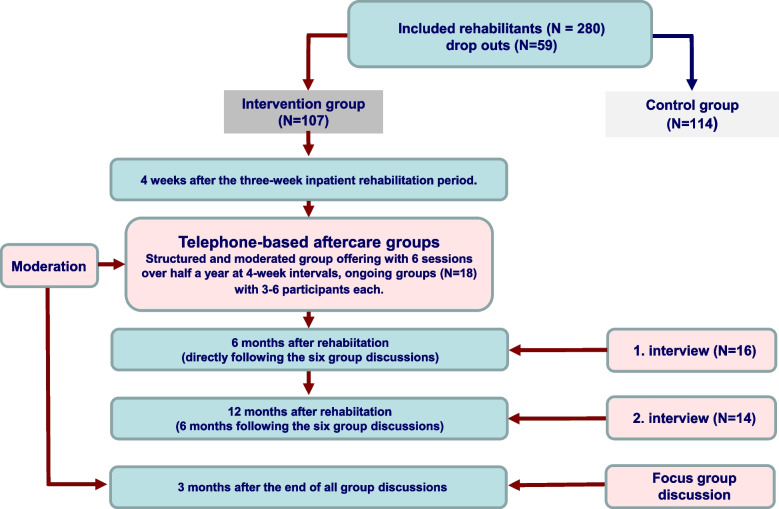
Procedure of the qualitative data collection (translated from [20]). Legend: *N* Number, *SGB* Social Security Code

The qualitative process data were collected by means of two longitudinal retrospective guideline-based telephone interviews with family carers of the intervention group after the end of the aftercare group in which they had participated and a telephone-based focus group interview with the two moderators at the end of the entire intervention period (see Fig. 3).

1. ***Longitudinal interviews.*** Both longitudinal interviews were conducted by telephone by one of the moderators of the aftercare groups (SL) and digitally recorded. The experiences of the family carers with the aftercare offering at the end of the intervention and 6 months later were surveyed (see Fig. 3). The guided telephone-based interviews included questions on the participants’ acceptance and satisfaction. They each began with a narrative introductory sequence related to the aftercare services. The implementation was oriented towards the focused interview [22] (for the guidelines of the first longitudinal interviews, see Additional file 3; for the guidelines of the follow-up interview, see Additional file 4).

The aim was to obtain a basis for interpreting these data in parallel with the quantitative data of the effects evaluation [10]. According to Flick [23], the design of the guideline and the focused interview should take into account the criterion of not influencing the interviewees, the specificity of the point of view, the coverage of broad understanding and the appropriate depth and personal reference for the interviewee [20].

(2) ***Focus group interview.*** The focus group interview with the two moderators was also conducted using guidelines (for the interview guidelines, see Additional file 5: Table A1) with a narrative entry sequence. It was conducted as a telephone conference by MB and RD and was digitally recorded [20]. Among other things, it contained questions about the acceptability of the groups and the accompanying folder or the feedback forms, the supervision of the moderators and the further development and optimization of the telephone-based aftercare service.

### Sample

All consecutive rehabilitants who in the course of a year, accompanied by a relative with dementia, were newly admitted to AMEOS Rehab Clinical Centre Ratzeburg, a rehab clinic for family carers, were addressed as potential study participants. Exclusion criteria were the presence of a personality disorder, psychotic symptoms, language barriers and cognitive impairments [11].

All newly arriving rehabilitants at the clinic were screened by the study staff on site regarding their eligibility for study participation according to the inclusion/ exclusion criteria. Eligible rehabilitants were then scheduled to attend the information session via the therapy control centre. During the information session, the rehab patients were informed about the study project and asked for their consent to participate in the study [10].

#### Quantitative process data

All family carers who were assigned to the intervention group (telephone-based aftercare group) in the randomized controlled longitudinal study (effect study) [10] (see Fig. 3) provided quantitative process evaluation data. In addition, the two moderators of the aftercare groups generated quantitative process data as part of the preparation of the memory protocols for the respective sessions of the telephone-based aftercare groups (see above).

#### Qualitative process data

Sixteen participants from the first four fully implemented aftercare groups with different characteristics of the variables of age, education, and care burden (defined by the care level of the relative with dementia) were selected and asked to participate in two longitudinal telephone-based interviews at six-month intervals after completion of the six-month intervention (see Fig. 3). Thereby care burden is defined by the care level as determined when assessing a person’s need for long-term care in the context of German long-term care insurance. In this context, the degree of care (of which there are five levels) is operationalized via the degree of independence in carrying out the activities of daily living. A higher care level of the relative with dementia means less independence and thus a higher care burden (or the other way around).

We used a purposeful sampling strategy with the aim to select cases with maximum variation for the purpose of documenting unique or diverse variations that have emerged in adapting to different conditions and to identify important common patterns that cut across variations [24]. Therefore, the aim of the sample selection was to obtain a heterogeneous sample with respect to the categorized variables of age, education, and care burden, so that all variable expressions (high vs. low) are present at least once in all categories. This maximum variation with regard to the variable expressions was given in sufficient form after the selection of 16 study participants.

The telephone-based focus group interview was conducted with the two moderators of the aftercare groups (see Fig. 3) [20].

### Data analysis

#### Quantitative process data

After the end of the intervention period, the feedback sheets and memory protocols were finally analysed. Quantitative data from the memory protocols and feedback sheets were analysed descriptively (frequencies and proportions, means, and measures of dispersion) using IBM SPSS Statistics, version 22.0. Individual items from the questionnaires were scored using five-point Likert scales. Due to the small cell frequency of the first and last category, the categories “very important” and “important” as well as “less important” and “unimportant” were combined into one category each.

#### Qualitative process data

Both longitudinal interviews and the focus group interview were transcribed with the program F4, oriented to the transcription rules according to Dresing and Pehl [25] and Kuckartz [26]. For the method of the focused interview with the predefined group (participants of the telephone-based groups or moderators) and the recording of their subjective views (acceptance and satisfaction with regard to the telephone-based aftercare groups, summarizing assessment of the aftercare offer), an evaluation via a coding procedure, such as the content-structuring qualitative analysis, seemed to make sense. This form of analysis is widespread in qualitative research, and different procedures and variants are described in the literature [27–29]. Qualitative content analysis as a rule-guided method should meet the criteria of object adequacy, reliability, unidimensionality, mutual exclusivity and exhaustiveness as requirements for the category system [26, 30]. The analysis of the interviews and the focus group discussion was therefore based on the qualitative content structuring analysis according to Kuckartz [26] using MAXQDA, version 2018.2. Kuckartz [26] describes this in seven phases: the initiating text work, the development of the main categories, the coding of the material on the basis of the main categories, the compilation of all text passages of the same main categories, the inductive determination of subcategories on the material, the coding of the material with the differentiated category system, and the analysis and visualization of the results. Since the interviews refer to concrete questions, only relevant text passages were selected for coding, according to the segmentation as described by Mayring [28]. To achieve high reliability and validity, intercoder coding was performed [20].

## Results

### Quantitative process data

#### Sample characteristics

A total of 107 family carers aged 52 to 86 years participated in the aftercare groups during the intervention period from August 2018 to March 2020 (for more detailed information on sample characteristics, see Table 1 of the publication on the results of the effect evaluation [10]) and provided quantitative process data (feedback sheets) in this context. Eighty-three individuals participated in all six scheduled group sessions. For organizational reasons, three groups were merged because some participants dropped out of the study immediately before or during the intervention. Ultimately, only 18 of the 21 originally planned aftercare groups were conducted. Each group had a minimum of three and a maximum of six participants. The two moderators who created the memory protocols for the telephone-based aftercare sessions were both trained social workers.

**Table 1:** Sample characteristics of the participants in the qualitative interviews (*N* = 16) (translated from [20])

№	sex	age	school-leaving qualification	person to be cared for	care level of the person to be cared for	age (categorized)^a^	burden of care (categorized)^b^	level of education (categorized)^c^
1	female	52	secondary school	husband	3	low	low	*high*
2	female	67	secondary school	mother	4	low	*high*	*high*
3	female	55	Abitur	mother	4	low	*high*	*high*
4	male	79	Abitur	wife	3	*high*	low	*high*
5	male	76	main school	wife	4	*high*	*high*	low
6	female	78	secondary school	husband	4	*high*	*high*	*high*
7	male	75	Abitur	wife	5	*high*	*high*	*high*
8	female	53	Abitur	father	4	low	*high*	*high*
9	female	69	main school	husband	3	low	low	low
10	male	80	main school	wife	4	*high*	*high*	low
11	male	83	main school	wife	4	*high*	*high*	low
12	female	66	Abitur	husband	4	low	*high*	*high*
13	female	82	main school	husband	2	*high*	low	low
14	female	81	main school	husband	4	*high*	*high*	low
15	female	65	secondary school	husband	3	low	low	*high*
16	female	78	secondary school	husband	4	low	*high*	*high*

^a^ = 50–69 years = "low”, from 70 Jahren = "high”

^b^ = care level 1 to 3 = "low”, care level 4 to 5 = "high”

^c^ = no school-leaving qualification, main school-leaving certificate = "low”, secondary school leaving certificate to Abitur = "high”

#### Memory protocols

The technical quality of the telephone-based aftercare groups was generally rated as good; there was an intermittent disturbance due to reverberation during the call in only five group sessions. Participants were disconnected in up to a quarter of the groups conducted, but this was usually resolved immediately. In 15% of the group sessions, there were short breaks due to technical problems or delays in access to the telephone conference. The formal procedure of the telephone-based groups was adhered to in almost all cases (95%). In the view of the moderators, the topics addressed in the respective sessions were of interest to all participants, and the content was communicated well. In exceptional cases, there were questions that could not be answered (2%, or *N* = 7); these were about very individual problems in dealing with the relative with dementia.

**Fig. 4:**
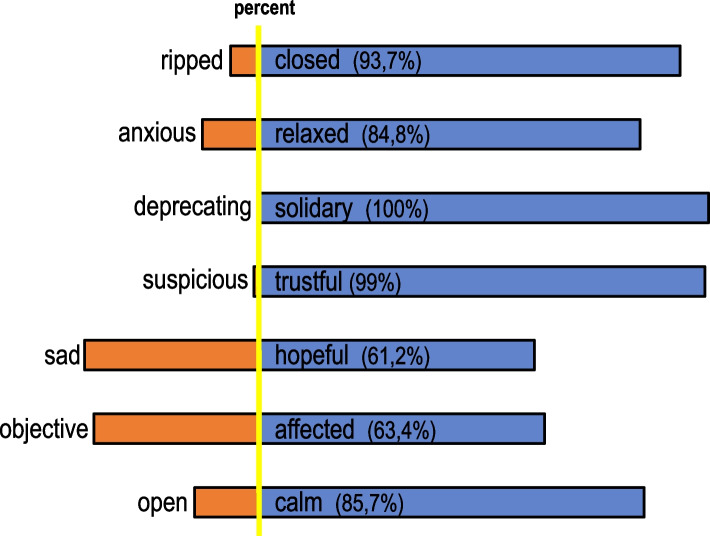
Atmosphere in the group

The mood in the groups was consistently rated as positive by the moderators, and the atmosphere was predominantly trusting, calm and relaxed (see Fig. 4). Depending on the topic of the group session, grief played a major role and was palpable in the atmosphere.

#### Feedback sheets

The feedback sheets were used regularly and could thus be directly incorporated into the formative process of the aftercare groups. Across all six group sessions, the range of the number of returned feedback sheets was between N = 63 and N = 73. The overall response rate of the feedback sheets was 82% (409 of 498 feedback sheets were returned by the participants).

**Fig. 5:**
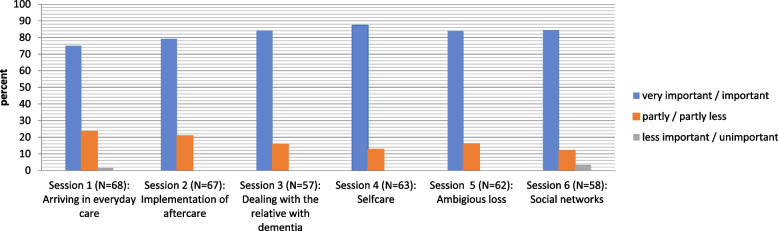
Relevance of the group theme. Legend: *N* = Number

The topics covered in the aftercare groups were rated as highly relevant (see Fig. 5). The topic of self-care achieved the highest positive rating. Only the introductory group session and the topic of building social networks were perceived as unimportant by one and two people, respectively. Almost all participants confirmed that they were able to contribute satisfactorily to the respective group topic. Two persons each denied this for the topic of the implementation of aftercare recommendations and the topic of self-care.

**Fig. 6:**
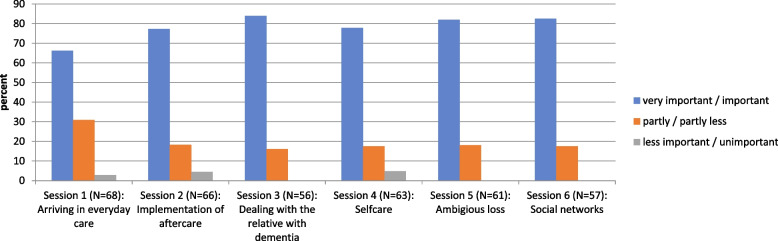
Relief through the group discussion. Legend: *N* Number

With the exception of two people, the majority of participants felt comfortable to very comfortable in the aftercare groups. Most family carers found the group discussion a relief, with the topics of dealing with the family member with dementia, building social networks and dealing with grief and loss being the most important (see Fig. 6).

The moderation was consistently and mostly positively evaluated. All interviewed persons rated the moderators with the given criteria as friendly, non-dominant and empathetic. The moderators were also described as confident and well prepared.

### Qualitative process data

#### Sample

As planned, 16 participants in the intervention group were recruited for the qualitative interviews (11 women and 5 men). The sample characteristics of all participating family carers are shown in Table 1. Six months later, at the second time point, only 14 individuals (9 women and 5 men) could be interviewed. One participant could no longer be reached using the original contact information, and another did not want to participate because her relative had died. The aim of the sample selection, which was to obtain a heterogeneous sample for the interviews with regard to the characteristics of gender, age, school-leaving qualification, relationship to the person to be cared for and care level of the person to be cared for (here care level is associated with care burden; for an explanation, see above section on sample/qualitative process data), was achieved since all characteristics (high vs. low) were present at least once in all categories. However, a higher care burden predominated for most of the participants.

#### First longitudinal interview

Six main themes emerged in the first semistructured longitudinal interviews with family carers in the intervention group, which had an average length of 19 min and were conducted between 1 and 3 weeks after t2: (1) “acceptability” consisted of the subthemes “group structure”, “group preparation”, “accompanying materials” and “participation organization”; (2) “group setting” consisted of the subthemes “group as a new experience”, “group exchange”, “knowing each other from rehab” and “moderation”; (3) “group contents” consisting of the subthemes “group themes”, “suggestions from the group” and “differentiation in the experience from other participants”; (4) “transfer to everyday care” consisted of the subthemes “personal insights and inner experiences”, “changed behaviour towards the person in need of care” and “implementation of the aftercare recommendations”; (5) “relatives' groups” consisted of the subthemes “previous experience with groups” and “attitude towards the use of relatives' groups”; and (6) “summary of the participants” consisted of the subthemes “situation of the participants after the telephone-based groups (6 months after rehab)”, “wishes and suggestions of the participants”, “positive comments about the aftercare groups” and “negative criticism of the aftercare groups”.

In the following paragraphs, these themes are summarized (for a more detailed description, see Additional file 6: Table A2).

1. ***"Acceptability".*** The overall picture shows that the telephone-based groups were well accepted, although participation for carers who were still working was partly hampered by time restrictions.
2. ***"Group setting"***. The exchange among the participants and the group setting met with great approval and were stated by the majority as pleasant, helpful, enriching and relevant for their own situation. The shared experience of caring for a person with dementia was seen as particularly supportive.
3. ***"Content of the groups"***. All group topics were considered interesting and relevant, especially the two topics on dealing with grief and loss in dementia and on sociolegal issues related to aftercare.
4. ***"Transfer to everyday care".*** In particular, the acceptance of dementia, the willingness to accept support, the recognition of one's own limits and a changed view of the care situation could be transferred by the family carers to their everyday care. In some cases, participation in the telephone-based aftercare group was also conducive to the use of, for example, outpatient care or support, day care and social legal advice as well as improving self-care.
5. ***"Support groups for family carers"***. Against the background of group experience in the telephone-based aftercare groups, most of the participants intended to look for a local family carer support group. Good professional support of the groups was of great importance for the carers. In this context, it was also important that adequate care was found for relatives with dementia during the time of participation in on-site support groups.
6. ***"Participants' summary"***. In retrospect, most of the participants found the aftercare offering to be a useful support built on the rehabilitation treatment. In particular, the exchanges in the group were experienced as positive. Both the continuation of the groups beyond the project duration and an adaptation of the aftercare groups to the special needs of, for example, working carers or children caring for their parents were conceivable for the participants as further developments of the groups.

#### Follow-up interview

Six main themes emerged in the semistructured follow-up interviews with family carers of the intervention group, which had an average length of 12 min and was between 1 and 3 weeks after t3: (1.) “content and subject matter” consisted of the subthemes “use of the accompanying materials” and “presence of the topics”, (2.) “transfer to everyday care” consisted of the subthemes “current care situation”, “personal insights and changed behaviour”, and “expansion of support services/utilization of assistance “, (3.) “group reference” consisted of the subthemes “exchange in the group”, “contact with the group”, “continuation of the groups” and “support groups for relatives” and (4.) “summary of the participants” consisted of the subthemes “personal criticism and suggestions” and “changed behaviour towards the person in need of care”.

In the following paragraphs, these themes are summarized (for a more detailed description, see Additional file 7: Table A3):

1. ***Content and subject matter.*** Six months after the end of the aftercare groups, most participants could no longer remember the specific content and topics of the groups, but they could remember the exchanges with the other group participants, which were perceived as helpful.
2. ***Transfer to everyday care.*** The burden of care and support had increased for all participants by the time of the second longitudinal interview, which, in most cases, was attributed to the progression of the dementia of the relative being cared for. Most participants continued to implement suggestions from the rehab and telephone-based groups six months after the end of the telephone-based aftercare groups, such as the use of day care or home adaptations.
3. ***Group reference.*** One year after the rehabilitation treatment, half of the participants were still in contact with other group participants and would have liked to continue the aftercare groups. Alternative participation in local relatives' groups was found to be useful but more difficult due to the distance and the need to organize the care of the relative with dementia during the groups.
4. ***Summary of the participants.*** Overall, the participants found the telephone-based aftercare service positive even six months after the end of the groups, especially with regard to the exchange of experiences and professional support. What was perceived negatively was the time feasibility. At the time of the follow-up interview, the topic "coping with grief and loss in dementia" was also perceived as particularly important, which is why it should be addressed more intensively in aftergroup sessions in the future.

#### Focus group interview

Five main themes emerged in the semistructured focus group interview with the two moderators of the telephone-based groups, which lasted 84 min and was conducted approximately three months after t3: (1.) “experience of the telephone-based aftercare groups” consisted of the subthemes “motivation of the participants” and “quality of the discussions”, (2.) “conducive structures and processes for successful implementation” consisted of the subthemes “set phone group appointments during the recruitment process”, “reminder call”, “use of the telephone to conduct the aftercare groups”, “structured groups”, “feedback sheets”, “supervision”, and “group folder”, (3.) “promoting and inhibiting factors for the implementation of the telephone-based groups” consisted of the subthemes “promoting factors” and “inhibiting factors”, (4.) “satisfaction of the participants with the aftercare offer” consisted of the subthemes "general satisfaction", "topics of group sessions", "implementation of aftercare recommendations", "perceived support from telephone-based aftercare groups", "perception as aftercare service," "frequency and length of sessions/length of aftercare measure overall”, "requests for optimization and change", "group folder" and "feedback sheets useful?" and (5.) "possibilities for improvement and further development" consisted of the subthemes "homogenization of groups", "number of group participants", "follow-up telephone-based aftercare group session", "continue telephone-based aftercare groups as open groups", "offer telephone-based aftercare groups as an aftercare service of the German Pension Insurance", "conduct aftercare groups as WhatsApp groups" and "conduct aftercare groups as video conferencing, e.g., Skype".

In the following paragraphs, these themes are summarized (for a more detailed description, see Additional file 8: Table A4):

1. ***Experience of the telephone-based aftercare groups.*** Moderating groups conducted by telephone is no easier or more difficult than conducting face-to-face groups, but it does require a special sensitivity in dealing with the participants. In the telephone-based groups, it is just as easy to get a group discussion going as in a face-to-face group, but the moderators experience the group discussions as more concentrated than in a face-to-face group.
2. ***Conducive structures and processes for successful implementation.*** In principle, the telephone was very well suited for conducting the aftercare groups. From an organizational point of view, reminder calls before the next group session were just as useful as setting the group dates early on during the phase of recruiting participants.
  The impulse lecture at the beginning of the groups by the moderators, the accompanying folder and the feedback sheets proved to be useful for structuring and preparing both the participants and the moderators for the telephone-based aftercare groups and were seen as very beneficial for the implementation of the groups. There was no need for professional supervision of the moderators during the implementation of the telephone-based aftercare groups, but supervision was perceived as helpful for the first implementation of the Talking Time REHAB aftercare groups.
3. ***Promoting and inhibiting factors for the implementation of telephone-based aftercare groups.*** For the organization of the aftercare groups, a binding schedule is obligatory. The implementation of the groups is hindered if the current problem situation of individual participants takes up too much space or if the care of the relative with dementia is not guaranteed during the telephone-based aftercare sessions. However, technical disturbances can hinder the implementation of the groups the most, such as interruptions of the conversation due to technical disturbances.
4. ***Participants’ satisfaction with the aftercare offer.*** Telephone-based aftercare groups can help to reduce the isolation of family carers and thus relieve them. Central to this is stable professional support and perceiving the group as supportive. The topics discussed were all considered relevant, especially the topic of coping with grief and loss in dementia ("ambiguous loss” [31]). In some cases, aftercare recommendations could be implemented with the support of the group or the moderator, such as organizing day care. For most participants, the duration and frequency of the aftercare measures and the number of five participants per group were correct. However, scheduling the groups in the evening could be more favourable for still-employed family carers, just as family members who care for their parents as children tend to prefer more homogeneous groups since the problems and issues of these carers may differ from those of carers of (married) partners. In this case, the content of the programme would also have to be adapted to the needs of working people.
5. ***Possibilities for improvement and further development.*** With some restrictions, it is conceivable to shorten the aftercare measure to four months and then to offer another single summarizing telephone-based group session after half a year.

The impulse lecture at the beginning of the groups by the moderators, the accompanying folder and the feedback sheets proved to be useful for structuring and preparing both the participants and the moderators for the telephone-based aftercare groups and were seen as very beneficial for the implementation of the groups. There was no need for professional supervision of the moderators during the implementation of the telephone-based aftercare groups, but supervision was perceived as helpful for the first implementation of the Talking Time REHAB aftercare groups.

An aftercare measure should be professionally guided and able to answer specific questions correctly and to accompany group dynamic processes. This is not offered by exchange platforms such as WhatsApp groups. In contrast, aftercare groups are conceivable as professionally moderated video conferences.

## Discussion

First, we would like to discuss the results of the present study within the larger project. These results confirm that the participants benefited from the aftercare offering and were able to integrate what they had learned in the rehab programme into their everyday lives. The collective experience in the group formed a resource and could support caring relatives in learning from each other and thus coping better with their everyday care and associated tasks. According to Yalom [32], this is a central therapeutic group effect factor. Here, the universal experience of suffering counteracts the social isolation experience of the individual and his or her individual fate and is thus perceived as unifying and strengthening. The participants confirmed that this positive and collective group experience was ultimately the main salutogenic benefit [33] of the telephone-based aftercare groups for the participants.

The exchange of information between family carers and learning from each other in the group in telephone-based aftercare groups can also help to build confidence in one's own ability to act and raise awareness of the need to accept external support. Studies show that family carers often make use of support only at a very late stage and take little care of themselves [3, 34]. At this point it should be critically noted that a transition to outpatient (family) groups could have been addressed more strongly during the aftercare group session to support the connection to care systems close to home.

The offer was well received, and the thematic structure seemed to meet the needs of family carers. Coping with grief and loss in dementia was seen as thematically very relevant by the family carers. In this context, the issue of ambiguous loss was specifically significant, with this phenomenon being described by Pauline Boss [31] as follows: although a person is still physically present, he or she is no longer truly mentally present. However, it also became apparent that there is an individual need for advice and support beyond the group topics addressed that could not be provided within the framework of the telephone-based aftercare groups, such as the search for a local facility or the need for psychotherapeutic advice.

After completion of the intervention, the information provided by the family carers could be used to derive possible adjustments to the content of the aftercare offering. For example, an adaptation of the groups could be conceivable for still-employed carers and would make sense as a preventive support offer. As a nationally representative study showed, the participation of carers in working life decreased with the progression of the dementia and one in five carers gave up their employment in the course of caring due to the high stress levels [35].

Now that we have just discussed the results of the present project internally, we would like to discuss in the following text the significance of the results external to the project and in relation to other study projects. It is certainly the case that many of our findings have already been shown by other studies in this productive research field. For example, a review paper by Lee and colleagues [36] found that in recent years, different technologies (internet, telephone) have been increasingly used to deliver social support interventions (individual or group format) and that these worked well and were able to reduce the burden experienced by family carers [37–41]. Of these reviewed studies, the technology-based social support interventions conducted in a group format [38–41] partly showed that similar or the same processes were at work in the social support interventions as those identified in this study. For example, the therapeutic group effect factor of the universality of suffering, which we have confirmed as a central effect factor of group interventions (see above), also plays a central role in these other technology-based social support interventions. However, the aspect in which the telephone-based aftercare groups compared to this intervention primarily differs is the use of a technology-based social support intervention as an instrument of rehab aftercare. Using a group intervention as a rehab aftercare measure is, to our knowledge, completely novel, certainly for Germany. The advantage of this technology-based and therefore location-independent group format is that the participants already know each other from rehab and can stay in contact as a group after rehab. Therefore, the innovative idea was to use the mutual learning and the mutual exchange of experiences of the rehab patients to implement the rehab aftercare recommendations in everyday life. What the results of the present process evaluation show, in addition to the effect evaluation of the project [10], is that TCI moderated exchange of experiences is really what helps to implement the aftercare recommendations in one's own everyday life and thus to stabilize the rehab effects. We know from an observational study which examined the sustainability of the effects of the rehab facility for family carers of people with dementia [9], who also participated in Talking time REHAB, that this stabilisation would definitely not have succeeded without the present aftercare intervention. Thus, this telephone-based aftercare group intervention added a completely new and, above all, effective intervention format [10] to the field of rehab aftercare, which in our view can be transferred to other settings (e.g., rehabilitation for other patient groups or their relatives (e.g., multiple sclerosis)) without greater difficulty.

### Strengths and limitations of the study

The central strength of this process evaluation is that a heterogeneous subsample with maximum variation regarding the variable expression was formed. This purposeful sampling strategy ensures representativeness and diversity of the family carers and therefore increases the significance of the data. On the other hand, we did not know a priori if the selected and categorized variables would really achieve the sampling of information-rich informants that cover the range of variation and if, therefore, an iterative approach of sampling and re-sampling to draw an appropriate sample, e.g., according to the grounded theory [42], would have led to clearer patterns. Another limitation may be that the feedback sheets were self-developed questionnaires and not questionnaires already validated by previous studies. However, many of the single items included in the questionnaires had been successfully applied in similar studies.

## Conclusions

Telephone-based aftercare groups for family carers of people with dementia are a useful and practical instrument in the context of rehab aftercare and can be conducted with little effort. They offer temporary support after rehab treatment and can help to deepen the learned knowledge, to ask thematic questions and to provid prepared aftercare recommendations during their implementation. This is particularly important in view of the requirements of the health insurance funds' discharge management (framework contract on discharge management in medical according rehab to the German Social Code V). The telephone-based setting was particularly accessible to the age group over sixty, since in Germany, unlike a computer, a telephone is available in virtually every household in this age group and the use of one is familiar. On the whole, it can be said that the telephone-based groups are very well accepted, the group effect factor supports the positive experience and learning from the other family carers in the group can provide longer professional support through the implementation of this aftercare offer.

## Supplementary Information

**Additional file 1.**

**Additional file 2.**

**Additional file 3.**

**Additional file 4. **

**Additional file 5.**

**Additional file 6.**

**Additional file 7.**

**Additional file 8.**

## Data Availability

The datasets used and/or analysed during the current study are available from the corresponding author on reasonable request.

## Abbreviations

ISE: Institute for Social Medicine and Epidemiology
MRC: Medical Research Council
N: Number
Rehab: Rehabilitation
REACH II: Resources for Enhancing Alzheimer’s Caregiver Health II
SPSS: Statistical Package for the Social Sciences
TCI: Theme-Centred Interaction
