# Supplementary material for: Telephone-based aftercare groups for family carers of people with dementia – results of a mixed-methods process evaluation of a randomized controlled trial
Source: BMC Health Serv Res. 2023 Jun 15;23:643. doi: 10.1186/s12913-023-09579-1 (PMC10273544; doi:10.1186/s12913-023-09579-1)
Supplement: Supplementary file 1 — Additional file 1. [file 12913_2023_9579_MOESM1_ESM.pdf]

1. Group details

1.1 group ID: ____________________

1.2 date: ____________________

1.3 duration: from _________ o'clock until _________ o'clock

1.4 moderator: ____________________

1.5 theme: ____

2. Participation details

No.:	Participant's ID	Participation:	If no or partially, were there known reasons?
2.1	________________	☐ yes ☐ no ☐ partially	______________________________
2.2	________________	☐ yes ☐ no ☐ partially	______________________________
2.3	________________	☐ yes ☐ no ☐ partially	______________________________
2.4	________________	☐ yes ☐ no ☐ partially	______________________________
2.5	________________	☐ yes ☐ no ☐ partially	______________________________

3. Assessment of the session

3.1 Technical assessment:

3.1.1 Was the voice quality good?

☐ yes

☐ no → please explain: ____

3.1.2 Were there any disconnections?

☐ no

☐ yes → please explain: ____

3.1.3 Were there delays or pauses due to technical defects?

☐ no

☐ yes → please explain: ____

3.1.4 Other?

____

3.2 Assessment of the process and the contents

3.2.1 Could the planned formal procedure be adhered to?

☐ yes
☐ no → please explain:

3.2.2 Topics addressed by the group participants in the discussion part

3.2.3 From the moderator's point of view, was the topic of interest to all group participants?

☐ yes
☐ no → please explain:

3.2.4 From the moderator's point of view, were the contents conveyed?

☐ yes
☐ no → please explain:

3.2.5 Were there any questions in the round that remained unanswered or could not be addressed further?

☐ no
☐ yes→ Which?

4. Assessment of the group

4.1 Were there any conspicuous features in the group dynamics? (For example, were there particularly influential or withdrawn participants? Were there particular dynamics around specific themes or issues?)

☐ no
☐ yes → in relation to:

4.2 Were there any problems for individual participants to get involved?

☐ no
☐ yes → please explain:

4.3 Group atmosphere

a)	restless	①	②	③	④	⑤	⑥	⑦	calm
b)	objective	①	②	③	④	⑤	⑥	⑦	affective
c)	sad	①	②	③	④	⑤	⑥	⑦	hopeful
d)	suspicious	①	②	③	④	⑤	⑥	⑦	trustful
e)	deprecating	①	②	③	④	⑤	⑥	⑦	solidary
f)	anxious	①	②	③	④	⑤	⑥	⑦	relaxed
g)	open	①	②	③	④	⑤	⑥	⑦	closed

5. Self-reflective assessment of the moderator

5.1 Were there any problems for the facilitator in running the group? If so, which ones? (e.g., in conveying the contents, in the group dynamics)

5.2 Other/comments for the next session (e.g., adjustments to the process and content, what should be addressed again in the next session)
