# Supplementary material for: Telephone-based aftercare groups for family carers of people with dementia – results of a mixed-methods process evaluation of a randomized controlled trial
Source: BMC Health Serv Res. 2023 Jun 15;23:643. doi: 10.1186/s12913-023-09579-1 (PMC10273544; doi:10.1186/s12913-023-09579-1)
Supplement: Supplementary file 2 — Additional file 2. [file 12913_2023_9579_MOESM2_ESM.pdf]

Additional file 2. Blank form of the feedback sheet

cipher: [ ]

Talking time REHAB feedback sheet

Dear Group Participant,

Please take a few minutes now, directly after our group session, to complete this form and return it to us. It will help us prepare for the next session and is an important component of our study.
You will find the stamped envelope at the end of your accompanying folder.

Thank you for your feedback!

1. Organizational details

1.1 Date of the telephone conference: ________________________

1.2 Were you able to attend the meeting as planned?

- ☐ yes
- ☐ no → **please continue from number 5**

2. Feedback on contents

2.1 How relevant or important did you find the theme of today's session with regard to your individual situation?

☐	☐	☐	☐	☐
--very important	important	partly/partly	less important	unimportant

2.2 Do you have any open questions about today's content?

- ☐ no
- ☐ yes→ which?

2.3 What was particularly important or helpful for you in today's session?

3. Feedback on your own experience in the group

3.1 From your point of view, were you able to contribute satisfactorily to the group discussion?

☐ yes
☐ no → why not?

3.2 How comfortable did you feel as a participant in the group session?

☐	☐	☐
very well	appropriate for the situation	rather uncomfortable

If you felt rather uncomfortable, are there reasons you would like to share with us?

3.3 How relieving did you find the group discussion?

☐	☐	☐	☐	☐
very relieving	relieving	neither nor	rather not relieving	not at all relieving

4. Feedback on moderation

4.1 I found the moderation to be (multiple answers are possible):

☐ helpful ☐ competent ☐ appropriate to the situation
☐ relieving ☐ friendly ☐ approachable

☐ unfriendly ☐ uncertain ☐ too dominant
☐ inappropriate ☐ poor prepared ☐ not empathetic

5. feedback on the aftercare recommendations

5.1 I have already succeeded in implementing some recommendations from the rehabilitation

☐ no
☐ yes → which?

5.2 I have a concrete need for support

… ☐ no
☐ yes → whereby?

6. Preparation for the next group meeting

6.1 Are there any themes that should be taken up in the next group meeting?

☐ no
☐ yes → which?

6.2 Do you have any further comments or questions?

☐ no
☐ yes → which?
