# Supplementary material for: Telephone-based aftercare groups for family carers of people with dementia – results of a mixed-methods process evaluation of a randomized controlled trial
Source: BMC Health Serv Res. 2023 Jun 15;23:643. doi: 10.1186/s12913-023-09579-1 (PMC10273544; doi:10.1186/s12913-023-09579-1)
Supplement: Supplementary file 3 — Additional file 3. [file 12913_2023_9579_MOESM3_ESM.pdf]

Additional file 3. Guidelines for the first longitudinal interview

Interview guidelines

Preprotocol (information can be taken from the available documents and does not have to be requested)

General data/information:

- Age
- Gender
- Care level of the person in need of care
- Family relationship to the person in need of care (spouse, father, mother, etc.)
- Level of education

Introductory question:

In the meantime, six telephone follow-up interviews have taken place in your group. Please tell us how you feel now after these group sessions.

Questions about the themes/contents of the groups:

- How did you find the topics of the group sessions?
- Were there any topics that particularly appealed to you or topics that you found less interesting or important?
- Would you have liked other themes for the group session? If yes, which ones?
- In your opinion, were the themes of the group session sufficiently dealt with?

Questions about the transfer to everyday care:

- Are there any changes in your daily care routine that you associate with the telephone-based aftercare groups of Talking Time REHAB? If yes, which ones?
- Can you describe moments when you were able to apply what you learned in the telephone-based aftercare groups or in the rehab? Which recommendations did you find particularly helpful?
- Has anything changed in your relationship or contact with your relative in need of care that you would relate to the telephone-based groups? If so, what?
- Has your environment, i.e., friends or family, noticed any changes in you in the last six months?

Questions about the group setting:

- How did you feel about the exchange in the group?
- Was the exchange helpful? If so, can you tell us where exactly, or where not?
- Were you able to accept or implement the suggestions of the group members? If so, what were they?
- Did you succeed in bringing your personal questions and concerns into the group? Can you give examples of this? If not, why were you not able to contribute?
- Was the exchange in the group a new experience for you or did you already have experience with groups (if yes, which ones)?
- After your experience with Talking Time REHAB, would you also attend an outpatient group for carers?

Practicability/acceptability questions:

- How did you feel about the practical feasibility of the phone calls? Do you see any barriers to implementation? Which ones?
- To what extent did you have to organise yourself to be present at the telephone group meeting?
- Were you able to prepare for the telephone groups on a regular basis? If yes, to what extent? If no, what prevented you from doing so?
- Were the accompanying folder and the materials helpful from your point of view? What would you have liked to have seen differently?

Summing up:

- Do you have any suggestions for improvement for the telephone-based groups?
- Is there anything you particularly liked or disliked about the telephone groups?
- How would you explain the telephone-based groups and what you have experienced to other carers?

Thank you for your participation and openness in the conversation!
