# Supplementary material for: Telephone-based aftercare groups for family carers of people with dementia – results of a mixed-methods process evaluation of a randomized controlled trial
Source: BMC Health Serv Res. 2023 Jun 15;23:643. doi: 10.1186/s12913-023-09579-1 (PMC10273544; doi:10.1186/s12913-023-09579-1)
Supplement: Supplementary file 4 — Additional file 4. [file 12913_2023_9579_MOESM4_ESM.pdf]

Interview guide (6 months after the telephone groups)

Preprotocol information can be taken from the available documents and does not need to be asked for.

General data/information:

- Age
- Gender
- Family relationship to the person in need of care (spouse, father, mother, etc.)
- Level of education

The following should be asked in advance:

- Degree of care (as it may have changed)
- Whether the person in need of care is still at home, in inpatient care, or has already died

Introductory question:

Dear Mrs/Mr..., the telephone groups were held about half a year ago. Looking back, how do you feel about this offer?

Themes/contents of the groups:

- Which topics or themes from the telephone groups are still present for you?
- Are there any topics or themes from the groups that still have a lasting effect or play a role for you and your relative? Can you give an example?

Questions about the transfer to everyday care:

- What has changed in your daily care routine?
- Are there any changes in your daily care routine that you associate with the rehabilitation or the telephone groups of Talking Time-REHAB? If yes, which ones?
- Were you able to continue implementing the suggestions discussed in rehab at home? What were they?
- Has anything changed in your relationship or contact with your relative in need of care that you would relate to the telephone groups? If yes, what?

Family carer support groups

- What is your attitude towards family groups? Has it changed after the telephone groups?
- Would you say that the exchange in the telephone group made you look for a relatives' group yourself?

Contact with the group

- Do you still have contact with the group? Do you still exchange information with each other?
- If yes, would you have maintained this contact even without the group, e.g., because you knew the person from rehab?
- If no, can you say why not? (e.g., no interest, no time...)

Summary

- Do you still use the folder/documents from Redezeit-Reha? If yes, in which way?
- In retrospect, would you have wished for more telephone-based support beyond the telephone groups? If so, for what reasons?
- Is there anything else you would like to add?

Thank you very much for your participation and openness in the interview!
