# Supplementary material for: Telephone-based aftercare groups for family carers of people with dementia – results of a mixed-methods process evaluation of a randomized controlled trial
Source: BMC Health Serv Res. 2023 Jun 15;23:643. doi: 10.1186/s12913-023-09579-1 (PMC10273544; doi:10.1186/s12913-023-09579-1)
Supplement: Supplementary file 5 — Additional file 5. [file 12913_2023_9579_MOESM5_ESM.pdf]

Additional file 5: Table A1. Guidelines for the focus group interview

Questions	Theme
MB: Introduction with reference to the speaking time project or DE-REACH. Again, brief derivation of the topics? How do the basic theoretical assumptions flow into the discussion facilitation?<br><br>In the following, the main topics are listed according to their application: practicability and further development.	Introduction
First of all:<br>What activities and tasks do you take on in the rehabilitation clinic?<br>How long have you been working in rehabilitation and specifically in this clinic?<br>How long have you been working with family carers?	Vocational background
1) How did you experience the moderation of the aftercare groups via the medium of telephone in general and in contrast to face-to-face groups?<br>• What are the differences compared to face-to-face groups?<br>• What are the advantages and disadvantages of conducting the groups by telephone in terms of moderation? What is difficult, and what is perhaps even easier?<br>• How was the quality of the discussions? How did you manage to create a balance between topics, i.e., in the sense of a topic-centred interaction?<br>*2) Which structures and processes contribute to a successful implementation of the aftercare offer?*<br>• Technique<br>• Was a reminder call always done? Was it practicable? Was it suitable to prepare for the next group?<br>• Timing of telephone-based groups<br>*3) What facilitating factors or what obstacles influence successful implementation?*<br>• Situation of the person with dementia<br>• Time aspects<br>• "Desire" aspects or motivational aspects<br>• How suitable did the participants find the topics dealt with/discussed? Are there any topics that the participants would have liked to have discussed additionally or alternatively?<br>*4) How satisfied are the participants with individual aspects of the aftercare services?*<br>• What aftercare recommendations did the participants receive at the end of rehab? How big are the differences between the rehabilitation participants? Are the aftercare recommendations at the end of rehabilitation reflected in the telephone-based groups? With what weight?<br>• Not all groups are rated equally well. When were the participants less satisfied?<br>• Not all participants contribute equally to the conversation, which is an indicator of discomfort in the group. How was this dealt with?<br>• How many participants dropped out of the telephone groups prematurely? Why?<br>• What findings and indications for optimisation have emerged from the personal interviews?	Practicability

4) What is the general assessment of the telephone groups in the context of rehabilitation?<br>• Composition and size of the groups? (e.g., differences in dementia severity, occupation)<br>• Extent and content of the units?<br>• What are the problems with changing group participants (merging groups)?	
Did it make sense to integrate the accompanying folder as an intervention component in the aftercare offer?<br>• Did the participants use the folder regularly for preparation and for their own benefit? How was this recorded?<br>• Patients' evaluation of the folder? How did they comment on it (too much, too complicated,…)?<br>• Were the feedback sheets evaluated by the participants? Can they be omitted in a possible transfer to standard care?<br>• Was the accompanying folder for the moderators helpful? What was the extent of its use?	Accompanying folder<br>feedback sheets
Did the professional supervision improve the quality of the implementation of the aftercare service at all? If so, how?<br>• By whom and how?<br>• What was discussed? What was the content?<br>• How often?<br>• Results and benefits?	Supervision
Where are there possibilities for improvement or further development of the telephone-based aftercare service?<br>• Size of the groups (time and staff)?<br>• Composition of the groups, fit (e.g., homogeneity of the groups with regard to occupation)?<br>• Contents (e.g., some topics not relevant)? Alternative: Ask participants in the clinics what they want to work on after rehab.<br>• Suggestion to participants to meet outside the telephone-based groups?<br>• Open groups conceivable (without moderator)? Prepare for themes in the rehab facility and exchange contacts?	Further development and optimisation
